# 

**Published:** 1899-05-20

**Authors:** 


					cs
E HOSPITAL. " Tne standard of highest purity."? Lancet, May 20th, 1899.
CADBURY'S K* CADBURY'S <-
COCOA \% M M COCOA
ABSOLUTELY PURE. NO DRUGS OR ALKALIES USED.
mms
? WESTERN INFIRMARY
No. ssoMvoi. XXVI. [Sw?!]
Saturday, May 20th, 1899.
r===*NORFOLK amd NORWICH H&8PITJLL
[S.bSortp,i??Te?,,] pRICE TWOpElfCE

				

